# Rational Fabrication of Functionally-Graded Surfaces for Biological and Biomedical Applications

**DOI:** 10.1021/accountsmr.4c00186

**Published:** 2024-09-29

**Authors:** Tong Wu, Xiaoran Li, Jiajia Xue, Younan Xia

**Affiliations:** † Medical Research Center, The Affiliated Hospital of Qingdao University, Qingdao University, Qingdao 266000, P. R. China; ‡ Innovation Center for Textile Science and Technology, Donghua University, Shanghai 201620, P. R. China; § Beijing Laboratory of Biomedical Materials, State Key Laboratory of Organic−Inorganic Composites, Beijing University of Chemical Technology, Beijing 100029, P. R. China; & The Wallace H. Coulter Department of Biomedical Engineering, Georgia Institute of Technology and Emory University, Atlanta, Georgia 30332, United States; ¶ School of Chemistry and Biochemistry, School of Chemical and Biomolecular Engineering, Georgia Institute of Technology, Atlanta, Georgia 30332, United States

## Abstract

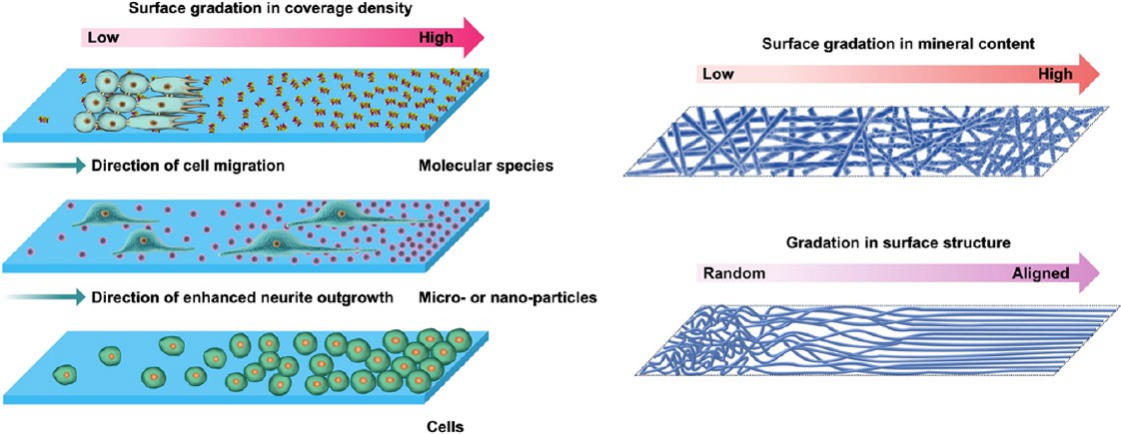

As a ubiquitous feature of the biological world, gradation, in either composition or structure, is essential to many functions and processes. Taking protein gradation as an example, it plays a pivotal role in the development and evolution of human bodies, including stimulation and direction of the outgrowth of peripheral nerves in a developing fetus. It is also critically involved in wound healing by attracting and guiding immune cells to the site of injury or infection. Another good example can be found in the tendon-to-bone enthesis that relies on gradations in composition, structure, and cell phenotype to create a gradual change in mechanical stiffness. It is these unique gradations that eliminate the high level of stress at the interface, enabling the effective transfer of mechanical load from tendon to bone. How to fabricate and utilize graded surfaces and materials has been a constant theme of research in the context of materials science, chemistry, cell biology, and biomedical engineering. In cell biology, for example, graded surfaces are employed to investigate the fundamental mechanisms related to embryo development and to elucidate cell behaviors under chemo-, hapto-, or mechano-taxis. Scaffolds based upon graded materials have also been widely explored to enhance tissue repair or regeneration by accelerating cell migration and/or controlling stem cell differentiation.

In this Account, we review our efforts in the fabrication and utilization of functionally graded surfaces. The gradation typically occurs as gradual changes in terms of composition, structure (e.g., pore size or fiber alignment), and/or coverage density of molecular species or larger objects such as particles and cells. Specifically, we focus on two strategies for generating various types of gradations along the surface of a substrate. In the first strategy, the substrate is vertically placed in a container, followed by the addition of a solution containing the functional component at a constant rate. Owing to the variations in contact time, the amount of the component deposited on the substrate naturally takes a gradual change along the vertical direction. In the second strategy, a moving collector or mask is used to control the amount of the component deposited on a substrate during jet printing or electrospray. As for applications, we highlight the following examples: (i) promotion of neurite outgrowth for peripheral nerve repair; (ii) acceleration of cell migration for wound closure; and (iii) mimicking of the structure and/or force transition at the tendon-to-bone enthesis for interfacial tissue engineering. The surface gradation can be presented in a uniaxial or radial fashion, and further integrated with the structural features on the underlying substrate to suit a specific application. In addition to general issues such as diversity of the surface gradation and reproducibility of the fabrication method, we also offer perspectives on new directions for future development. The systems and strategies discussed in this Account are expected to open the door to a range of fundamental inquires while enabling various biological and biomedical applications.

## 1 Introduction

**Figure 1:**
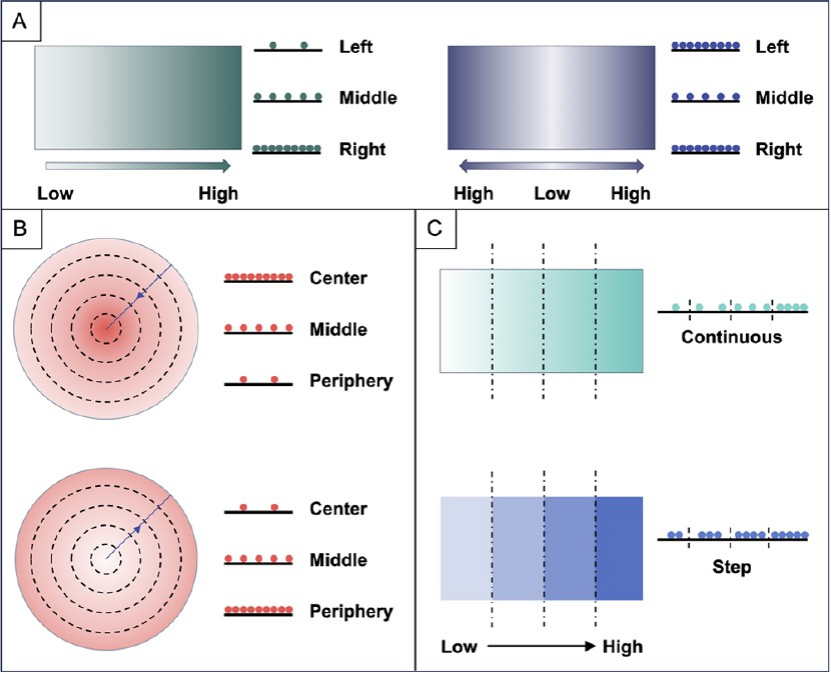
Schematic illustration showing the three major patterns of lateral gradation across a surface: (A) unidirectional and bidirectional, (B) circular, and (C) continuous or step function.

Gradation refers to the gradual changes of something over a distance, or continuous variations in the magnitude of a property along an axis.^1^ As a universal feature in biological systems, it plays vital roles in the development and physiology of tissues or organs.^2^ In general, gradation can take two different forms: lateral (across a surface) versus vertical (along the normal to a surface). Lateral gradation is primarily concerned with the two-dimensional arrangement of compositional or structural features on the surface of a substrate, as well as its use in manipulating the migration and differentiation of cells. In contrast, vertical gradation mainly involves the spatial variations of various types of features in the bulk of a three-dimensional construct. Given the pivotal role of lateral gradation in influencing cellular behaviors through the provision of direct contact guidance from compositional, structural and/or biochemical cues, here we focus on lateral gradations created on the surface of various types of substrates. Typically, surface gradation can be fabricated in three major patterns: unidirectional, bidirectional, and circular (Figure 1, A and B). The changes can be either continuous or as a step function (Figure 1C).

Organisms have ingeniously developed and utilized gradations in many different forms during the development and maturation of tissues or organs.^3,4^ Typical examples include morphogenic, compositional, structural, and mechanical variations, as well as gradations in cell phenotype and population; all of which play pivotal roles at different developmental stages of the organism.^5−7^ For example, molecular gradation along the longitudinal axis of the human hippocampus informs large-scale behavioral systems.^8^ The epithelial layer of skin contains gradation in porosity to act as a potential niche for cells and neovascularization. Architectural gradation, which manifests as differences in fiber orientation across cardiac tissue, and mechanical gradation at a load-bearing musculoskeletal interface (also known as the enthesis) are two additional examples. Specifically, the structural gradation associated with the muscular fibers in the cardiovascular system is known to accelerate cell proliferation and promote cell migration for the faster regeneration of cardiovascular tissue.^9−11^ The native interface connecting two dissimilar tissues typically embraces gradations in composition, architecture, and cell phenotype.^12,13^ Taking the tendon-bone enthesis as an example, it facilitates joint movement by leveraging gradations over four distinct zones from uncalcified tendon to calcified bone.^14,15^ The native gradations are constructed using localized variations in terms of chemical composition and/or constituent density, as well as structural characteristics, and they can manipulate the fates of stem cell by controlling their interactions with the environment.^3,16^ Following the concept of “biomimetics”, scaffolds capable of recapitulating the gradations existing in native tissues have found widespread use in biological and biomedical applications.

Inspired by the native gradations in biological systems, our research has centered on the development of scaffolding materials capable of reproducing the transitional gradients in native tissues. In this Account, we highlight our efforts in functionalizing solid substrates with surface gradations. We begin with two strategies that have been developed to fabricate graded surfaces, followed by a discussion on translating the gradations into biological effectors and biomaterials. Finally, we highlight a set of biological and biomedical applications enabled by the graded surfaces, including the promotion of neurite outgrowth, acceleration of collective cell migration, and repair or regeneration of graded connective tissues.

## 2 Fabrication of Functionally Graded Surfaces

**Figure 2:**
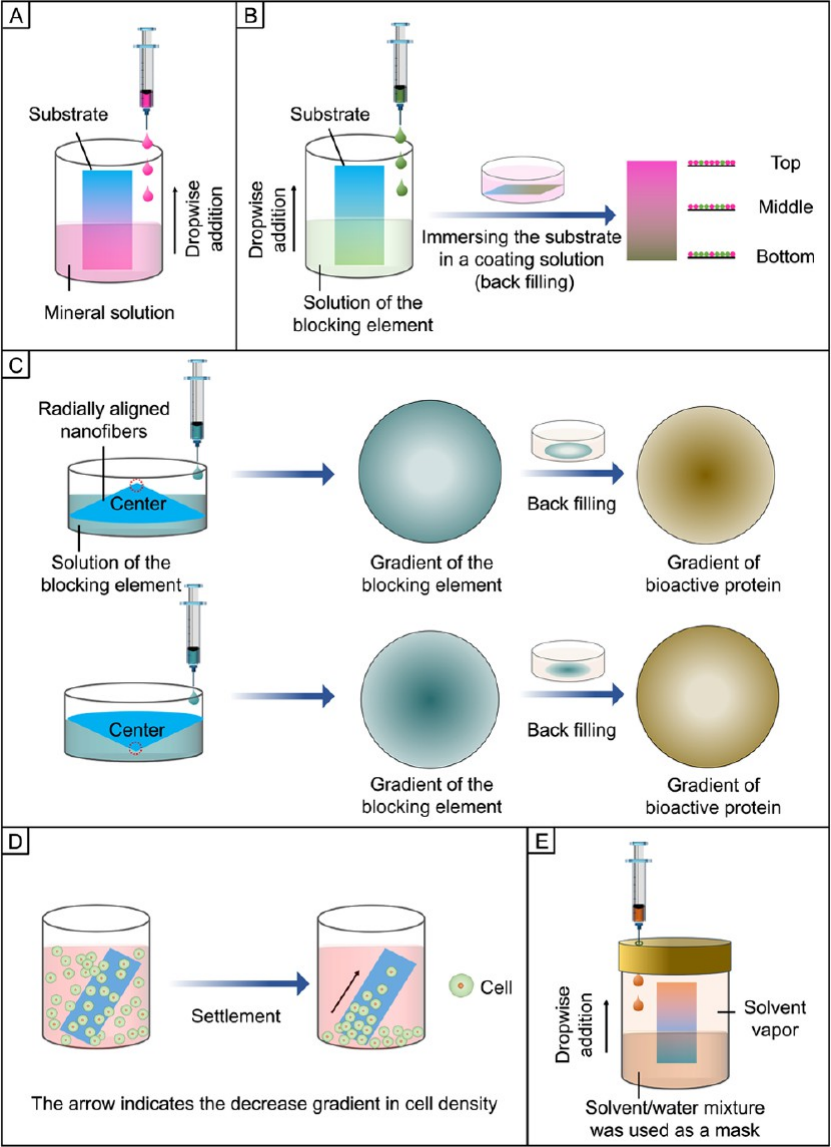
Manipulation of the contact time between the functional component and the surface of a substrate to create gradation. (A) Fabrication of linear gradation in mineral content. (B, C) Creation of (B) linear or (C) circular gradation in bioactive proteins with the use of a blocking element. (D) Generation of linear gradation in cell density. (E) Production of gradation in fiber alignment and/or porosity by controlling the exposure of a nanofiber mat to the vapor of a solvent.

**Figure 3:**
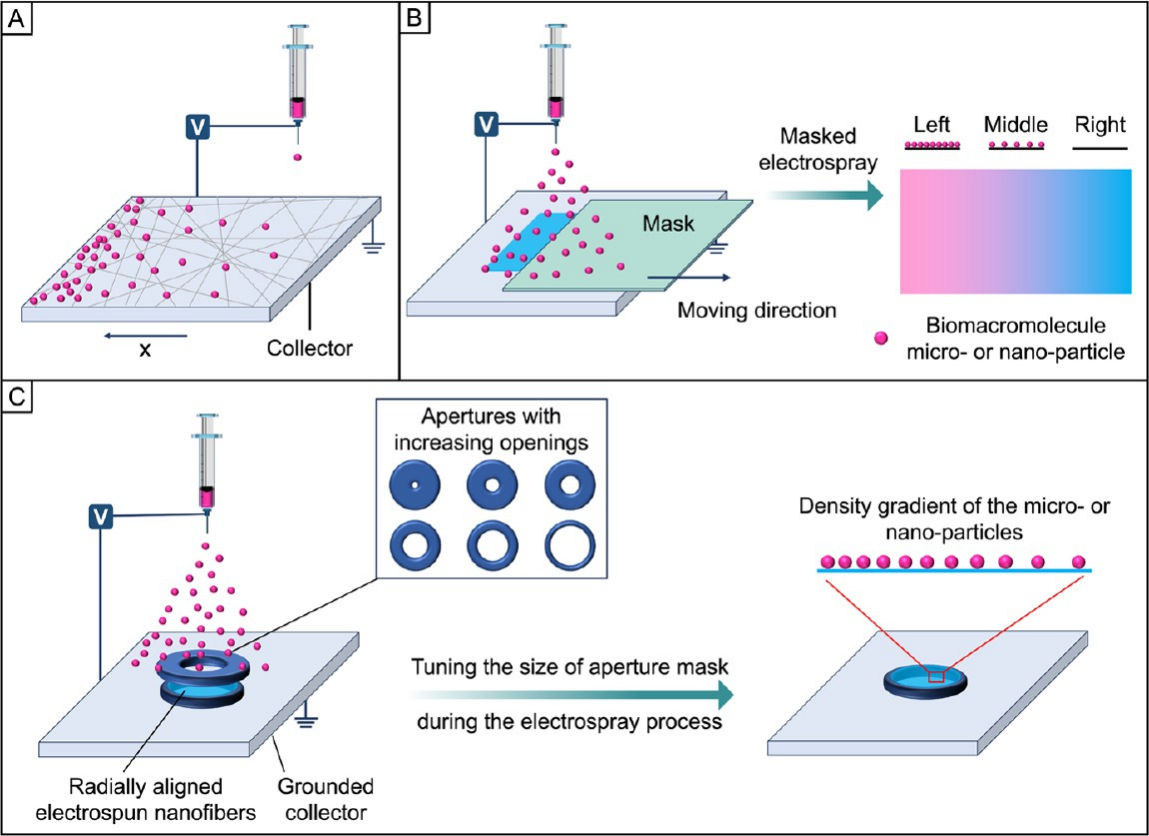
Modulation of the deposited amount of a functional component to generate a graded surface. (A, B) Generation of unidirectional gradation in the density of biomacromolecule micro- or nanoparticles on a substrate through (A) the use of a movable collector or (B) the introduction of a movable mask for electrospray. (C) Fabrication of biomacromolecule micro- or nanoparticles in radial gradation on a substrate by imposing a size-tunable aperture as the mask.

We have developed a number of methods for the fabrication of functionally graded surfaces. These methods can be broadly divided into two groups, with each group involving a different strategy. The essence is to impose a spatiotemporal modulation to the interaction between the functional component to be deposited and the surface of a substrate. In the first strategy, the contact time and thus the duration of deposition is regulated to create a functionally graded surface (Figure 2). In the second strategy, the amount of the functional component to be deposited is modulated by introducing a movable collector or mask (Figure 3). In both cases, the surface gradation can take a linear or circular pattern depending on the experimental setup. Both strategies can be adapted to accommodate different systems, including various functional components and substrates. In general, the first strategy is advantageous when applied to bioactive proteins or growth factors of low molecular weights in conjunction with a functionalized surface that can be stably immobilized with the bioactive components. However, this strategy is only suitable for generating the same continuous gradients on the top and bottom surfaces of a substrate. The second strategy, which is based on electrospray, allows for the generation of either continuous or step gradients with micro- or nanoparticles composed of biomacromolecules or polymers, or particles carrying the desired payloads. Such a gradation can provide not only the biological effect but also the contact cue resulting from the particle density. Moreover, the gradations on the top and bottom surfaces can differ from each other, and the gradients can take an either unidirectional or bidirectional pattern, allowing them to be readily tailored for specific applications. The sole restriction is the necessity for components that are suitable for electrospray.

### 2.1 Manipulation of the Contact Time

This strategy offers a simple and versatile route to generate linear or circular gradation in coverage density of a functional component that can be dissolved or dispersed in a solution by manipulating the contact time between the solution or suspension and the substrate.^17−20^ In a typical process, a substrate is placed in a container, to which a solution or suspension is added dropwise (Figure 2A). As such, the amount of the deposited solute or colloidal particles will naturally take gradation along a specific direction of the substrate. Depending on the nature of the functional component involved, it can be immobilized on the substrate via physical adsorption or chemical attachment. Furthermore, by varying the concentration and injection rate of the solution or suspension, as well as the tilt angle of the substrate, one can vary the slope of the gradient. This strategy works well for a variety of materials or systems. In an early demonstration, we fabricated scaffolds with gradation in mineral content along the uniaxial alignment direction of electrospun nanofibers to mimic the compositional and mechanical gradients typical of the tendon-to-bone enthesis.^17−19^

When applied to expensive proteins or growth factors, however, the unavoidable waste of a large amount of the solution becomes a major concern. To address this issue, we developed a variant that relies on the use of an inexpensive protein such as bovine serum albumin (BSA) as a blocking element to generate the gradation (Figure 2, B and C). Specifically, BSA is deposited on a surface in gradation, followed by backfilling the bare regions with the active protein (used at a very small amount) to generate a gradient opposite to that of BSA. This backfilling method has been used to generate surface gradients of protein-based growth factors, along the direction of alignment, on nonwoven mats of uniaxially- and radially aligned nanofibers, respectively.^21,22^

The capability of this strategy was also extended to fabricate graded surfaces featuring cellular and structural transitions.^20,23^ As shown in Figure 2D, one can insert a tilted substrate into a container filled with a homogeneous suspension of cells. Owing to the different volumes of cell suspension above the different regions on the substrate, the number of sedimented cells naturally forms a continuous gradient in cell density along the direction of insertion. By tuning the tilting angle of the substrate, one can control the slope of the gradient. Such a gradient in cell density was also created with the use of two different types of cells along the opposite directions on the same substrate (e.g., a nonwoven mat of nanofibers) to suit applications such as the repair or regeneration of interface tissues. In another system, gradual changes in surface structure were created due to the difference in swelling extent when poly(lactic-*co*-glycolic acid) (PLGA) nanofibers were exposed to ethanol solution or its vapor.^23^ It was found that PLGA nanofibers were swollen to become welded or overwelded when treated with ethanol molecules evaporated from the solution. As such, the nanofibers were quickly welded and even fused by ethanol vapor within 3–5 min, whereas they were not welded in ethanol solution for more than 30 min. The primary reason for this phenomenon is the slow motion of molecules in the solution phase due to hydrogen bonding between ethanol and water molecules. Ethanol molecules are unable to escape from the “cages” of water molecules without first breaking the hydrogen bonds. As a result of this dramatic difference in kinetics for welding the nanofibers, we were able to use the ethanol solution as a natural mask and gradually tune its volume and thus the masked area to control the exposure time of PLGA nanofibers to the ethanol vapor within a window of about 5 min (Figure 2E). In this way, a gradual change in surface structure from aligned to random nanofibers was formed, together with gradation in pore size for the resultant mat.

### 2.2 Modulation of the Deposited Amount

Surface gradation can also be fabricated by modulating the deposited amount of a functional component, and this strategy is often integrated with techniques such as electrohydrodynamic jet printing or electrospray to generate a surface gradient in particle density. For example, with the use of a movable collector, one can adjust its positions at different time points during the collecting process by setting its moving parameters in advance. As shown in Figure 3A, when a nozzle is placed on the top of the collector, the ejected solution or suspension is randomly deposited on the collector. As such, one can spatially adjust the deposited amount by controlling the moving speed of the collector. In one demonstration, a mat of collagen fibers was placed on a movable substrate to collect collagen-binding domain fused stromal cell-derived factor-1α (CBD-SDF1α) during electrohydrodynamic jet printing.^24^ When the moving speeds of the collector are adjusted in both *x* and *y* directions, both continuous and step gradients can be produced. Furthermore, it is feasible to manipulate the gradient profile by varying the concentration of CBD-SDF1α. This strategy can be extended to generate opposite gradients of two different types of functional components on the same surface by sequentially adjusting the collector movement.

As an electrohydrodynamic process, electrospray is widely used to produce particles made of different components such as polymers, biological effectors, a combination of them, or cells.^25−29^ In addition to controlling the movement of a collector and thus varying the coverage density of the electrosprayed particles, we introduce a physical mask to produce similar gradients. Figure 3, B and C, shows a schematic illustration of how to generate a graded coating of electrosprayed micro- or nanoparticles on the surface of a substrate with the use of a movable mask. In a typical process, the collector is fixed while a movable mask (e.g., a square substrate and a size-tunable aperture) is placed between the nozzle and collector to spatially tune the deposition time of the electrosprayed particles on different regions of the substrates. Using this method, we have fabricated gradients of microparticles made of PLGA or biomacromolecules on glass slides or nonwoven mats of uniaxially- and radially aligned nanofibers, respectively.^25,30−32^ This method is also capable of creating reverse or bidirectional gradients of different types of particles by controlling the movement of the mask. The formation of a graded coating can be reinforced by placing a movable magnet under the collector to facilitate the deposition of the electrosprayed particles on the desired regions.^32^ The electrosprayed particles can also be modified or preloaded with biological effectors to further expand their biofunction and thus augment their capability to control cell behaviors. This is often achieved by combining the contact guidance from topographic cues (i.e., density gradient of the particles) with the biological cues from the conjugated or encapsulated effectors. Such a masking method can be employed to directly generate gradients of functional components for various applications.

## 3 Functional Components for Creating Graded Surfaces

A variety of functional components can be used to create surface gradation through deposition and physical or chemical adsorption.^10,33^ Here we focus on typical components compatible with the two strategies discussed in Section 2, and the gradation is presented in the form of gradual changes in coverage density of molecular species, inorganic solids, particles, and cells, as well as structural transition in pores and alignment. Table 1 shows a summary of the functional components. Proteins, including various types of growth factors, represent a major class of molecular species that can be used to develop functionally graded surfaces by controlling the duration of contact time. In terms of inorganic solids, mineral content is often used to produce surface gradation by controlling the contact time between the mineral solution and the substrate. Such gradation can be integrated with aligned-to-random nanofibers to recapitulate the transition zone between tendon and bone. The gradation in coverage density of electrosprayed micro- or nanoparticles are usually generated by modulating the deposited amount of the particles. The graded surfaces can be fabricated using either biomacromolecules or polymers as the coating materials. Cell density and porosity or fiber alignment have also been used to create graded surfaces by manipulating the contact time between the cell suspension or solvent vapor and the substrate. Except for the structural gradation that must be produced on nonwoven mats of uniaxially aligned nanofibers, all other types of gradients can be fabricated on glass slides or nonwoven mats of nanofibers featuring different fiber alignments and should be extendible to other types of substrates. In terms of the gradient profile, continuous changes are typically involved whereas gradation in molecular species and particles can also be created as step functions. In all cases, the slope of the gradient is tunable depending on the contact time or deposited amount.

**Table 1:** Summary of the Functional Components That Have Been Explored to Create Graded Surfaces

Type of gradation		Substrate	Strategy for creating the gradation	Pattern of gradation	References
Molecular species	Bioactive molecules such as proteins or growth factors	Glass slides	Manipulation of the contact time or modulation of the deposited amount	Continuous or step-function	(21, 22, 24)
		Nonwoven mats of nanofibers			
Inorganic solids	Mineral	Nonwoven mats of nanofibers	Manipulation of the contact time	Continuous	(18, 19, 35)
Particles	Biomacromolecules	Nonwoven mats of nanofibers	Modulation of the deposited amount	Continuous or step-function	(25, 30−32)
	Polymers	Glass slides			
Cells	Cells	Glass slides	Manipulation of the contact time	Continuous	(20)
Surface structures	Porosity	Nonwoven mats of nanofibers	Manipulation of the contact time	Continuous	(23, 35)
	Alignment				

### 3.1 Molecular Species

**Figure 4:**
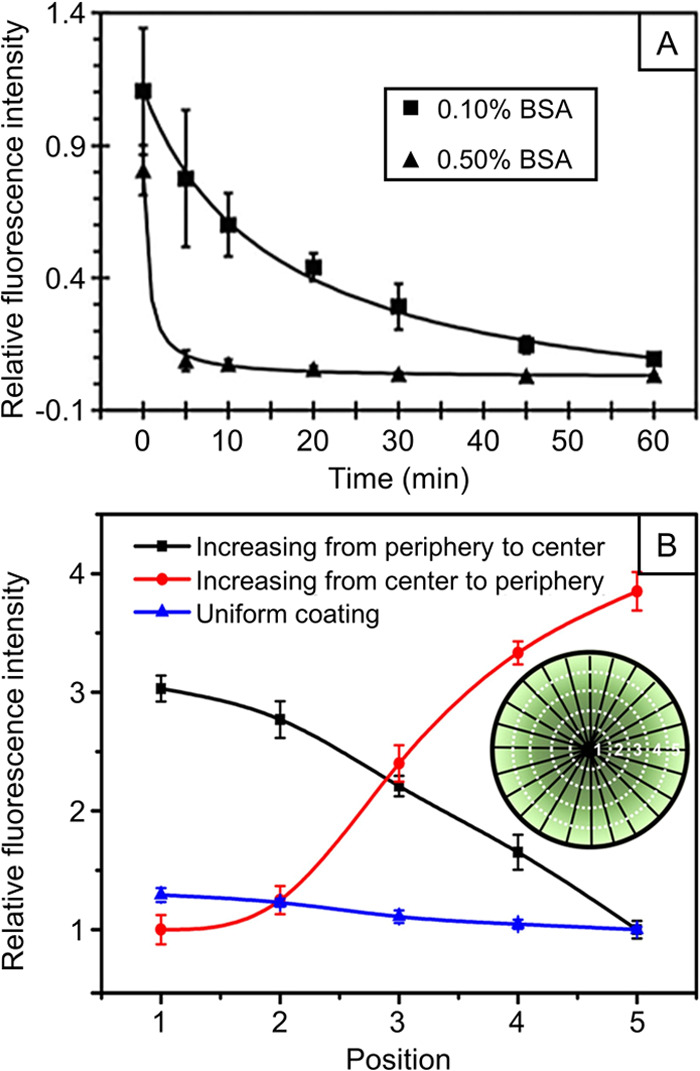
(A) Plots of relative fluorescence intensities for the FITC-BSA adsorbed on uniaxially aligned nanofibers as a function of immersion time in 0.1% and 0.5% BSA solutions, respectively. A linear gradient in FITC-BSA was achieved with the use of 0.1% BSA. (B) Plots of relative fluorescence intensities showing circular gradients of FITC-BSA increasing from the center to the periphery or along the opposite direction on radially aligned nanofibers. Adapted with permission from ref (21) (A) and ref (22) (B). Copyright 2017 Royal Society of Chemistry and 2018 American Chemical Society, respectively.

Surface gradation in terms of coverage density of molecular species (e.g., proteins or growth factors) has been extensively explored, and it has found use in a broad range of applications. As a typical example, using the strategy illustrated in Figure 2B, we have successfully fabricated linear gradation in fluorescein isothiocyanate-labeled bovine serum albumin (FITC-BSA) on nonwoven mats of uniaxially aligned poly(caprolactone) (PCL) nanofibers (Figure 4A).^21^ In another study involving the method shown in Figure 2C, we integrated a circular gradient of proteins or growth factors with radially aligned PCL nanofibers.^22^ As shown in Figure 4B, with FITC-BSA as a model, the gradients can be maneuvered to increase either from the periphery toward the center or *vice versa*. For the gradient increasing from the center to the periphery, the fluorescence intensity in the peripheral region was nearly four-time stronger relative to that at the center (red line, Figure 4B). Similarly, the fluorescence intensity decreased by 3-fold from the center to the periphery for the gradient along an opposite direction (black line, Figure 4B). The decrease in fiber density from the center to the periphery also had an impact on the generation of protein gradients.^34^ In these studies, the FITC-BSA was also switched to a variety of proteins or growth factors such as nerve growth factor (NGF), laminin, and epidermal growth factor. By modulating the deposition time (Figure 3A), we also fabricated a graded layer of SDF1α coating on the surface of a nonwoven mat of electrospun collagen fibers.^24^ It was demonstrated that the gradation could be created in different patterns, including continuous changes with different slopes and step functions along the fiber alignment. These gradients are well-suited for use with surfaces containing oriented structures, such as nonwoven mats of uniaxially aligned nanofibers.

### 3.2 Inorganic Solids

**Figure 5:**
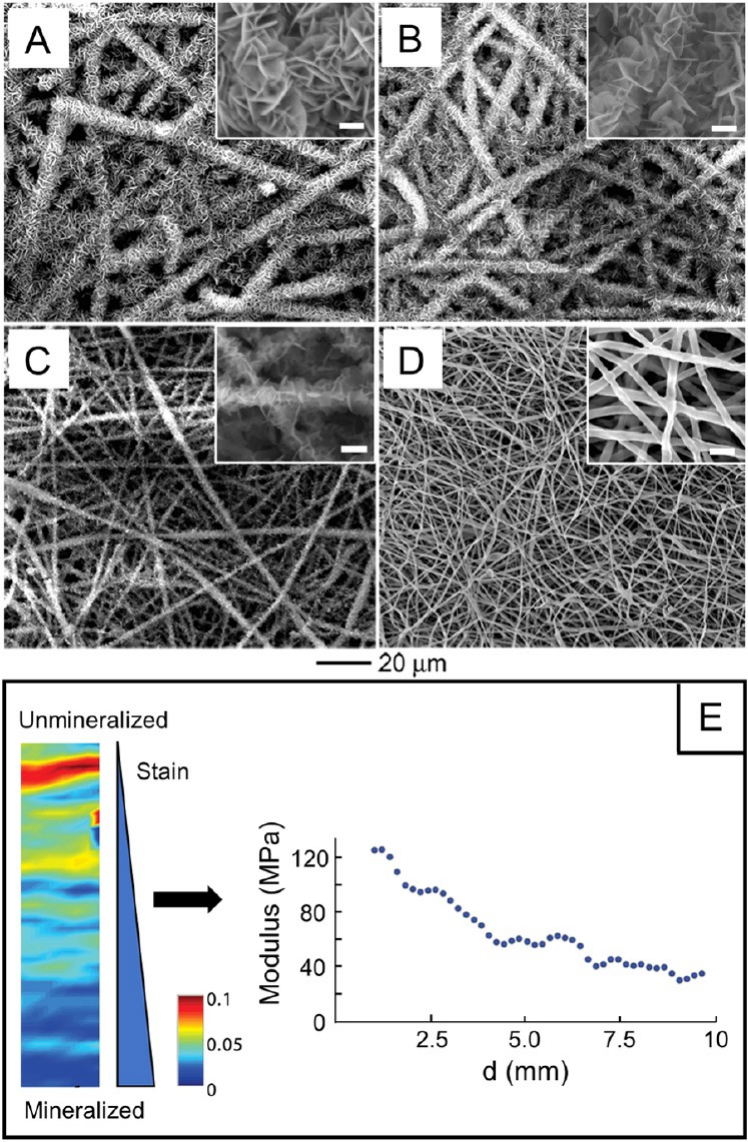
(A–D) SEM images of calcium phosphate coatings on a plasma-treated nonwoven mat of PLGA nanofibers. The images were taken from different regions, separated by (A) 0, (B) 6, (C) 9, and (D) 11 mm from the edge of the substrate with the highest mineral content. The scale bars in the insets are 2 μm. (E) Local strain fields presented using a heat map, together with a plot of the modulus changes. With decreasing calcium phosphate content, strain increased and it was the highest on the unmineralized side of the scaffold, whereas the Young’s modulus was the lowest. Reproduced with permission from ref (18). Copyright 2009 American Chemical Society.

With the use of a modified 10-fold-concentrated simulated body fluid (10SBF), calcium phosphate could be readily formed and deposited on the surface of electrospun nanofibers.^18,19^ The thickness of the mineral coating was dependent on the mineralization time, and it significantly affects the strength, toughness, and modulus of the nanofiber scaffolds. Furthermore, we successfully used the method shown in Figure 2A to fabricate scaffolds made of nonwoven mats of PLGA nanofibers, together with gradation in mineral content.^18,19^ As shown by the scanning electron microscopy (SEM) images, the nanofibers were covered by a thick mineral layer at one end of the scaffold (Figure 5A), and the mineral thickness gradually decreased with the distance farther away from the edge of the substrate with the highest mineral content (i.e., with the longest mineralization time), see Figure 5, B–D. As a result, we created an increasing gradient in modulus together with a decreasing gradient in strain along with the increase in mineral content (Figure 5E). The incorporation of inorganic components within a polymeric matrix is the primary factor responsible for the observed increase in modulus and decrease in tensile strain. The inorganic anchors restrict the movement of polymer chains, thereby contributing to the observed changes in mechanical properties. Such gradation can be further integrated with scaffolds featuring a structural change from aligned to random fibers to mimic the tendon-to-bone enthesis.^35^

### 3.3 Micro- and Nanoparticles

**Figure 6:**
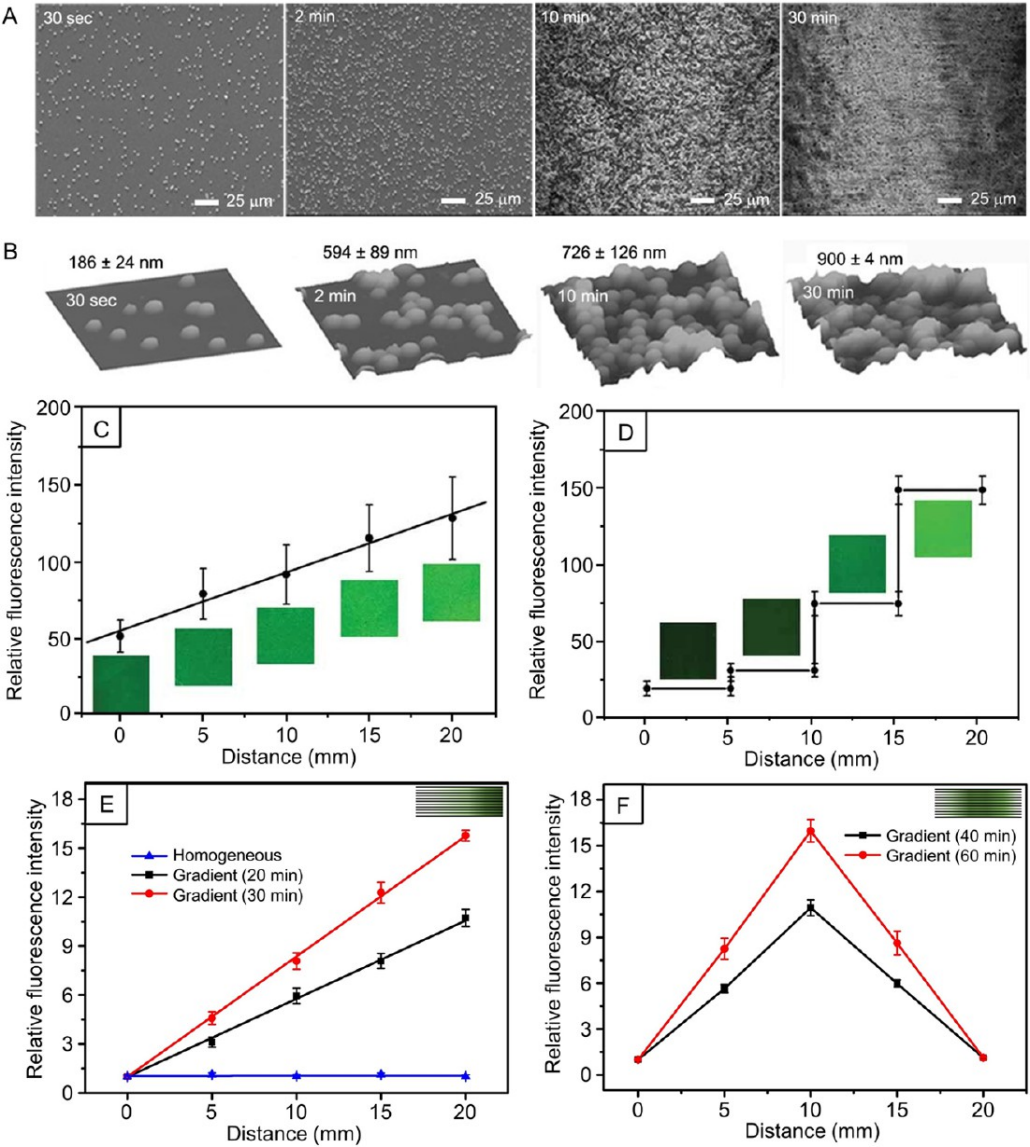
(A) SEM and (B) atomic force microscopy images of PLGA microparticles deposited on glass slides with different collection times. (C, D) Plots of relative fluorescence intensities separately showing (C) continuous and (D) step-function gradients of FITC-BSA-loaded PLGA microparticles on glass slides as a function of the distance away from the edge of the substrate having the lowest particle density. Typical fluorescence micrographs are presented at the corresponding regions in (C) and (D). (E) Unidirectional and (F) bidirectional gradients of electrosprayed FITC-BSA-loaded collagen nanoparticles deposited on uniaxially aligned PCL nanofibers, and the insets in (E) and (F) illustrate the corresponding gradients, respectively. Reproduced with permission from (A–D) ref (25) and (E and F) ref (30). Copyright 2010 Wiley-VCH and 2020 Wiley-VCH, respectively.

We have fabricated gradation on planar surfaces in terms of coverage density for particles made of either polymers or biomacromolecules.^25,30−32^ The fabrication relies on our ability to modulate the coverage density during the electrospray of particles (Figure 3, B and C). In one demonstration, we achieved a gradual increase in the coverage density of electrosprayed PLGA microparticles and the concomitant decrease in interparticle separation with the modulation of deposition time (Figure 6, A and B).^25^ The PLGA microparticles can be grafted with biochemical components or loaded with biological effectors to enhance their functionality. Using the method displayed in Figure 3B, we fabricated different types of density gradients (i.e., continuous versus step function) of FITC-BSA-encapsulated PLGA microparticles on glass slides (Figure 6, C and D). Such gradients not only provide topographic cues due to the surface roughness but also offer chemotaxis through the controlled release of a payload from the polymeric microparticles. We also directly used biomacromolecules for electrospray to generate unidirectional, bidirectional, and circular gradients on flat substrates or surfaces with specific structures (e.g., nonwoven mats of uniaxially- or radially aligned nanofibers).^30−32^ Taking FITC-BSA-loaded collagen particles as an example of biomacromolecules, we generated both unidirectional and bidirectional gradients in particle density with variable gradient slopes on nonwoven mats of uniaxially aligned PCL nanofibers (Figure 6, E and F).^30^ In another demonstration, we fabricated circular gradation of collagen nanoparticles on radially aligned PCL nanofibers using the setup shown in Figure 3C.^31^ The same gradients have also been successfully created by switching collagen to a combination of different types of biomacromolecules (e.g., a mixture of collagen and fibronectin or laminin) to suit different applications. Both the functional component used for electrospray and the collecting surface can be varied in response to the needs from the range of biomedical applications.

### 3.4 Live Cells

**Figure 7:**
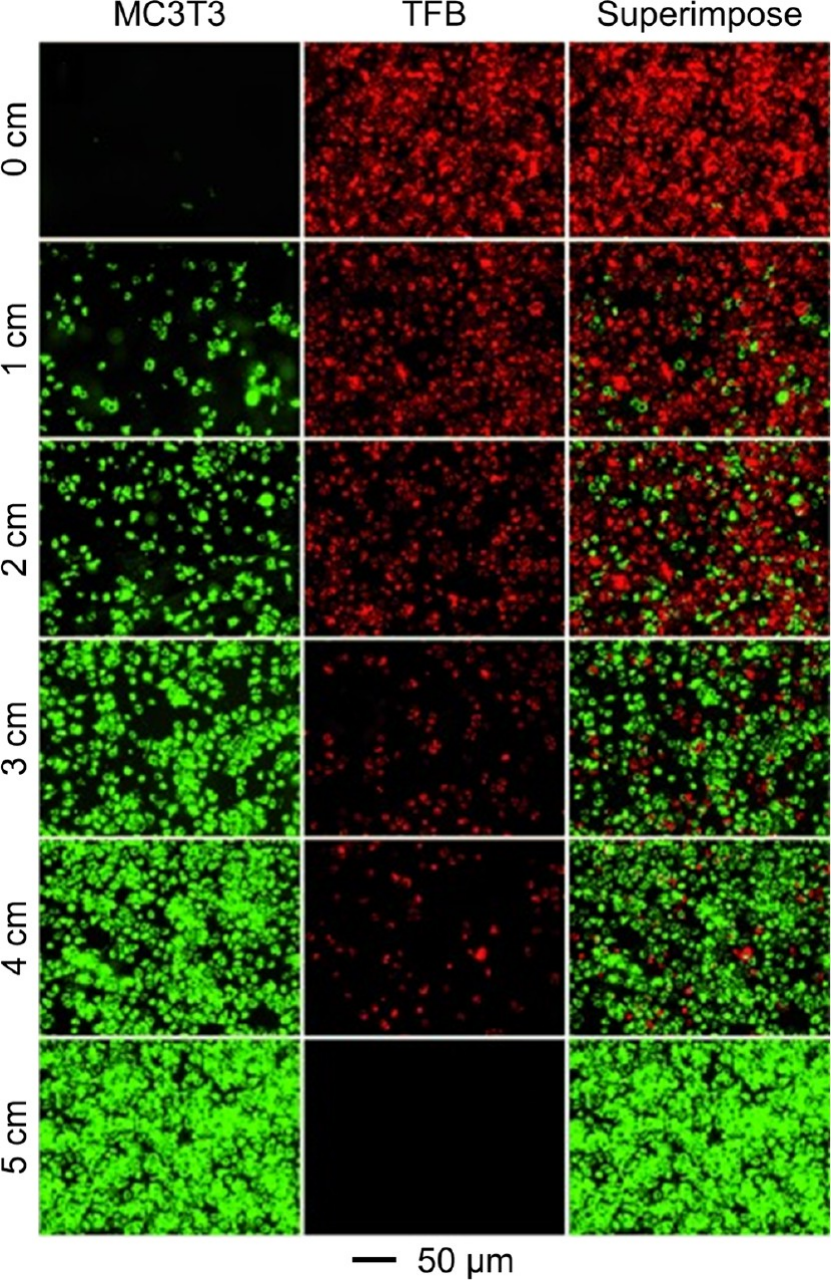
Fluorescence micrographs showing the opposite gradients in coverage density for MC3T3 preosteoblasts (green) and tendon fibroblasts (red) cells on a glass slide. Reprint with permission from ref (20). Copyright 2013 Wiley-VCH.

Live cells have also been used to fabricate gradation in cell type and/or cell density on the surface of a substrate. In a typical process, a glass slide was inserted into a homogeneous suspension of the cells at different tilting angles (Figure 2D).^20^ Due to the difference in volume and thus cell number above different regions on the slide, a gradient in the density of MC3T3 cells was created once the cells had sedimented and attached to the surface. The capability of this method was further extended to generate opposite gradients in terms of coverage density for DiO-labeled MC3T3 cells (green) and DiI-labeled tendon fibroblasts (red) on the same substrate (Figure 7). By varying the concentration of cells in the suspension, it is expected that the slope of the gradient can be varied in a controllable way. Such a spatial arrangement is expected to affect cell–cell interactions by producing gradation in extracellular matrix molecules during tissue repair.^36,37^

Live cells can be employed as the functional component to produce cellular gradients. Essentially all types of cells can be used to create the density gradation as long as the cells have the ability to adhere to the surface of the substrate. In the event of inadequate cell adhesion, the surface can be modified through the application of oxygen plasma treatment or biocoating. In general, such modification will improve the reproducibility of a fabrication process, as well as the stability of the resultant gradient.

### 3.5 Surface Structures

**Figure 8:**
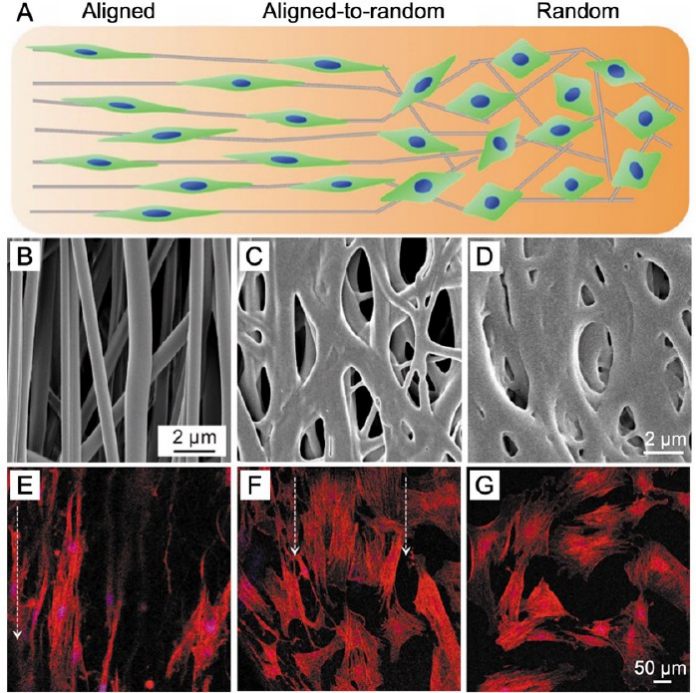
(A) Schematic illustration showing the transition of cell morphology when cultured on a surface made of aligned-to-random nanofibers to mimic the tendon-to-bone insertion site. (B–D) SEM images showing the structural gradation changing from (B) aligned to (D) random organization on a mat of PLGA nanofibers. (E–G) Fluorescence micrographs of bone marrow stem cells after culture on such a substrate for 3 days. The white arrows indicate the direction of fiber alignment. Reproduced with permission of ref (23). Copyright 2019 Wiley-VCH.

In addition to the gradients of physical objects, the surface itself can also be managed to form gradation in terms of structural features. We have developed a hierarchical surface capable of mimicking the tendon-to-bone enthesis by presenting gradual changes from uniaxially aligned nanofibers to random, overwelded nanofibers (Figure 8A). Moreover, the surface porosity changed from high to low along with the direction of aligned-to-random transition (Figure 8, B–D). The success of this fabrication relies on the control of the extent of welding by regulating the exposure of uniaxially aligned PLGA nanofibers to the vapor of a solvent (Figure 2E). The working mechanism can be attributed to the difference in motion capability exhibited by ethanol molecules in the vapor and solution phases, respectively. When cultured on such a surface, bone marrow stem cells exhibited highly organized morphologies at the “aligned” regions, random morphologies at the “random” areas, and graded transition in between (Figure 8, E–G).^23^ The morphological changes of the cells were consistent with those of the underlying nanofibers.

## 4 Biological and Biomedical Applications

Gradation in biomolecules and physical structures exists in many types of tissues and are essential to various biological functions and processes.^33,38^ With the creation of graded surfaces on planar substrates or nonwoven mats of nanofibers, we have developed a variety of model systems for biological and biomedical inquiries, including promotion of neurite outgrowth, acceleration of cell migration, engineering of interfacial tissues, and augmentation of wound healing.

### 4.1 Promotion of Neurite Outgrowth

**Figure 9:**
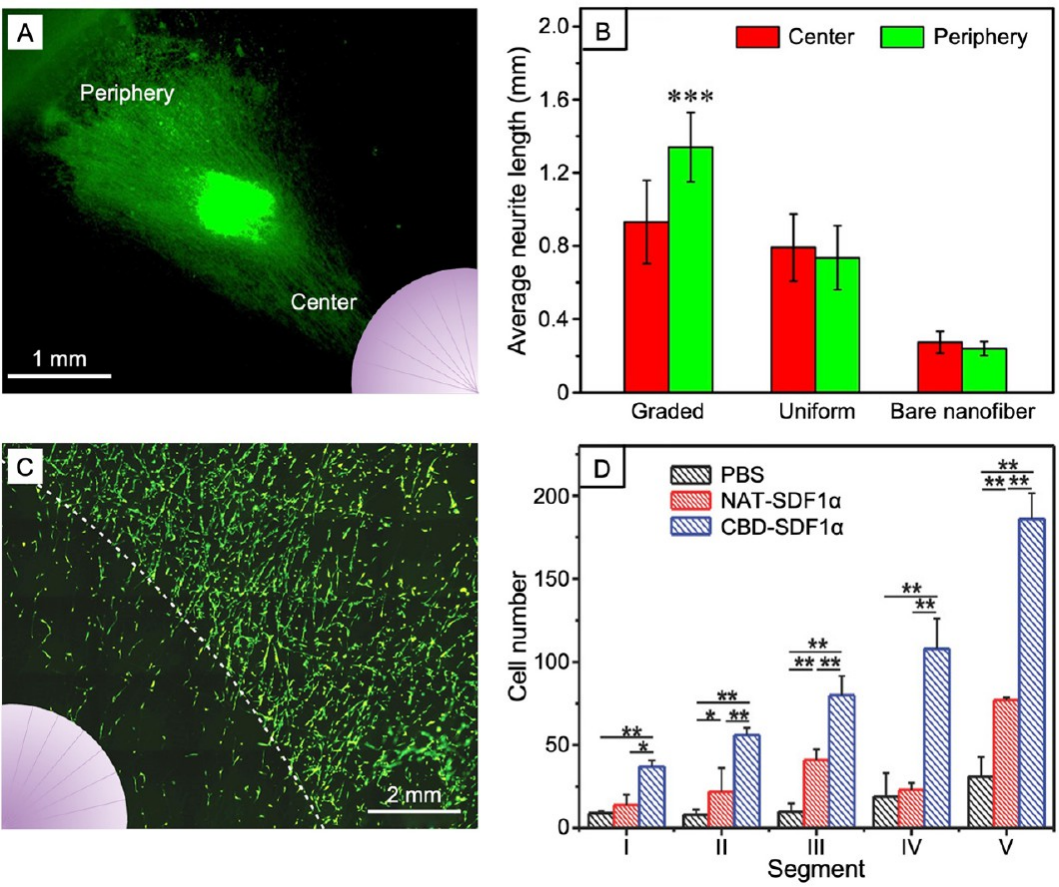
(A) Fluorescence micrograph showing neurite outgrowth from the DRG body on radially aligned nanofibers coated with a gradient of NGF increasing from the center to the periphery. (B) Comparison of the average neurite lengths outgrowing from the DRG bodies when cultured on bare nanofibers and nanofibers covered by a gradient or uniform coating of NGF. (C) Fluorescence micrograph showing the migration of neural stem cells on radially aligned nanofibers immobilized with CBD-SDF1α in gradation. (D) Cell numbers at different migration regions after separately culturing the cells on the nanofiber mats immobilized with CBD-SDF1α, native SDF1α (NAT-SDF1α), and PBS for 1 day. The insets in (A) and (C) show the pattern of surface gradation. Reproduced with permission from ref (22) (A and B) and ref (34) (C and D). Copyright 2018 American Chemical Society and 2016 Wiley-VCH, respectively.

During neuronal development, growth cones, which are located at the leading edges of neurites, play a vital role in sensing and responding to the microenvironmental cues for pathfinding.^39−41^ In addition to the contact guidance arising from the underneath substrate, chemoattraction is of great importance in guiding the outgrowth and extension of neurites. By decorating the surface with gradation in growth factor or density of electrosprayed particles, we developed a variety of graded scaffolds to augment neurite outgrowth. In one study, a circular gradient of NGF increasing from the center to the periphery was fabricated on a mat made of radially aligned PCL nanofibers.^22^ The direction of NGF gradient was consistent with the radial alignment of the fibers. Owing to the chemotaxis arising from the NGF gradient, the length of neurites extending from the dorsal root ganglion (DRG) body to the peripheral region (consistent with the increasing gradient) was 44% longer than those extending toward the central region (against the increasing gradient, Figure 9, A and B). Such a scaffold holds the promise for use in promoting the radial axon growth in retina.^42^ By depositing electrosprayed nanoparticles made of a mixture of collagen and laminin in a density gradient on a mat of uniaxially aligned PCL nanofibers, we also showed the enhancement of linear extension of neurites from DRG bodies.^30^ The surface gradation, in either bioactive molecules or particles, can be integrated with topographic guidance, for example, microfibers engraved with nanoscale grooves, to further augment the outgrowth and extension of neurites.^43^

### 4.2 Acceleration of Collective Cell Migration

Collective cell migration is central to many physiological and pathological processes, including embryonic development, immune response, cancer metastasis, and tissue repair or regeneration.^44−47^ It is well-established that the migration speed is dependent on the sensory integration between leader cells and extracellular cues, as well as the interaction between leader cells and follower cells.^48^ By creating a gradient of SDF1α, increasing from the periphery to the center, on a scaffold comprised of radially aligned PCL/collagen nanofibers, the migration of neural stem cells from the periphery to the central area was accelerated relative to the blank scaffold, regardless whether the SDF1α was fused with CBD before immobilization on the nanofibers (Figure 9, C and D).^24^ Similarly, we fabricated scaffolds capable of promoting the migration of NIH-3T3 fibroblasts and keratinocytes by coating the surface of radially aligned PCL nanofibers with laminin and epidermal growth factors, respectively, in a gradient increasing from the periphery to the center.^22^ Such graded surfaces provided a chemotaxis effect on the cells, leading to a major increase in cell number in the migration regions and improved migration index relative to the blank scaffold or a scaffold covered by a uniform coating of the same biological effector. When both sides of the same scaffold are presented with the graded surface, they are expected to find use in the regeneration of both epidermis and dermis tissues during wound healing. We also demonstrated the ability to accelerate the unidirectional migration of bone marrow stem cells and bidirectional migration of Schwann cells, respectively, using a surface with a density gradient in nanoparticles made of collagen or a blend of collagen and fibronectin.^30^ Altogether, these studies demonstrate that one can accelerate the collective migration of cells toward the intended site by introducing a surface gradient in the concentration of a soluble factor or the coverage density of electrosprayed particles.

### 4.3 Mimicking of the Tendon-to-Bone Enthesis

**Figure 10:**
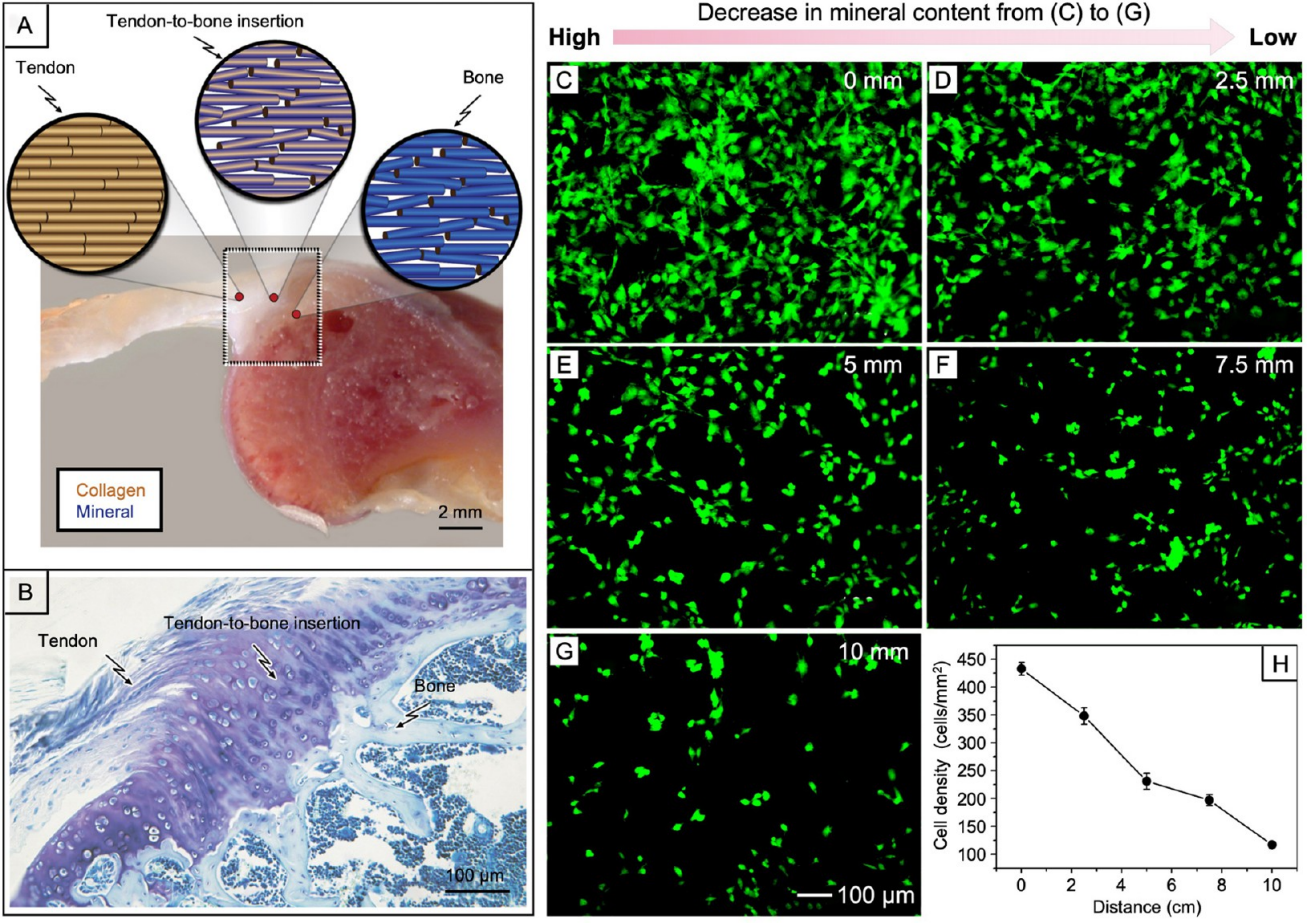
(A) Schematic illustration showing the graded transition of collagen fiber alignment and mineral content from a side view of a mouse supraspinatus tendon-to-bone enthesis. (B) Histologic observation with toluidine blue stain showing the tendon-to-bone insertion. (C–G) Fluorescence micrographs of the MC3T3 cells after culture for 3 days on a mat comprised of PCL nanofibers precoated with gelatin and then a graded mineral layer. The distance was measured from the edge of the substrate with the highest mineral content. (H) Average cell density along with the decrease in mineral content. (A, B) Reproduced with permission from ref (49). Copyright 2012 Taylor & Francis. (C–H) Adapted with permission from ref (18). Copyright 2009 American Chemical Society.

The native tendon-to-bone enthesis embraces a functionally graded transition from soft tissue to hard tissue, with a fibrocartilaginous interface in between (Figure 10, A and B).^49^ The gradation includes an aligned-to-random structural change for collagen fibers, a gradual increase in mineral content from tendon to bone, and thus a transition in mechanical strength over a range of more than two orders in magnitude. These graded transitions are impaired during injuries and can hardly be regenerated during natural healing or surgical repair. To reconstruct the tendon-to-bone enthesis, an essential prerequisite is to replicate and sustain these graded transitions together with the differentiation of associated cell populations. To this end, we have demonstrated the fabrication of a nanofiber-based scaffold containing both aligned and random regions to mimic the change in fiber orientation at the enthesis.^50^ The modulus and ultimate stress exhibited by the scaffold were found to be disparate in the aligned and random portions, together with a morphological transition for tendon fibroblasts. We also upgraded the scaffold by adding gradual changes in fiber alignment between the aligned and random regions, which could induce the same orientational transition for the morphologies of bone marrow stem cells (Figure 8).

We further demonstrated that gradation in mineral content on the surface of a scaffold made of electrospun nanofibers resulted in the creation of a gradient in mechanical stiffness, as well as cell activity.^18,19^ For example, when a nonwoven mat of PLGA nanofibers was coated with calcium phosphate in a linear gradient, it showed gradation in mechanical properties (i.e., localized strain and Young’s modulus) along the gradient.^18^ The deposited mineral contributed to the increase in strength and modulus of the nanofiber mat. When subjected to cell culture, MC3T3 cells showed a linear decrease in cell density along with the decrease in mineral content (Figure 10, C–H).

**Figure 11:**
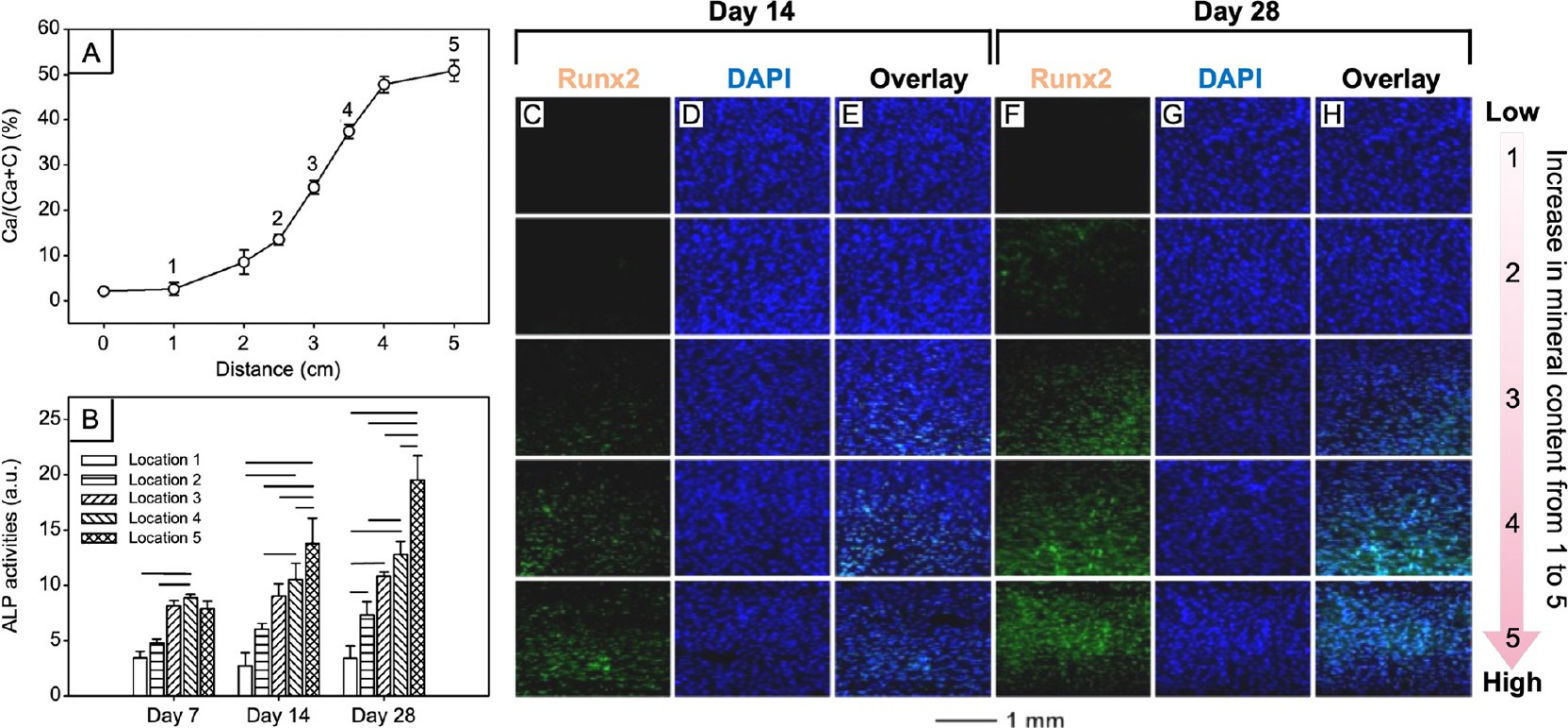
(A) Energy dispersive X-ray quantification showing the gradation in mineral content on a mat of nanofibers. The distance was measured from the edge of the substrate with the lowest mineral content. (B) Quantification of ALP activity after seeding ASCs on the graded surface for 7, 14, and 28 days. Locations 1 to 5 correspond to those marked in (A). Runx2 staining of ASCs seeded on aligned nanofibers with a spatial mineral gradient after culture for (C–E) 14 and (F–H) 28 days in proliferation medium. (C, F) Runx2 staining. (D, G) 4′,6-diamidino-2-phenylindole (DAPI) staining. (E, H) Overlaid images of Runx2 and DAPI staining. Runx2 staining was positively correlated with increasing mineral content and increased with culture time. Reproduced with permission from ref (19). Copyright 2014 American Chemical Society.

The osteogenic differentiation of stem cells also changed accordingly because of the gradient in mineral content. For example, a scaffold of PLGA nanofibers covered by a mineral coating in gradient facilitated the graded osteogenesis of adipose-derived mesenchymal stem cells (ASCs).^19^ As shown in Figure 11A, the mineral content increased monotonically over a 5–cm distance on the scaffold. When seeded with stem cells and cultured for 7, 14, and 28 days, respectively, we observed an increase in the expression of alkaline phosphatase (ALP, Figure 11B). In addition, the expression of runt-related transcription factor (Runx2, an early marker of osteoblast differentiation) was positively correlated with the mineral content (Figure 11, C–H). The gradation in osteogenesis can be attributed to the combined effect from both compositional gradation (i.e., mineral content) and mechanical signal (i.e., mechanical stiffness). These results indicate that the recapitulation of structural changes, cell phenotypes, and mineral gradient at the tendon-to-bone enthesis can all be achieved by coating a surface with a mineral in gradation. As a remaining challenge, it is still difficult to replicate the mechanical transition during enthesis repair, which is considered the most critical issue in ameliorating the interfacial stress concentration and thus facilitating force transmission between these two dissimilar tissues.

Over the past few years, the use of functionally graded surfaces has yielded encouraging outcomes in the promotion of neuronal growth, acceleration of collective cell migration, and augmentation of tendon-bone repair. It is anticipated that future applications of these graded surfaces and related materials will lead to clinical use in peripheral nerve repair, wound healing, and/or interfacial tissue engineering. In particular, as brain has a clear layered transition structure and exhibits structural variations and functional specificity across different regions, it is expected that functionally graded surfaces and materials may offer promising applications in the domain of brain injury repair. However, since the structure and function of the brain are complex, extensive research needs to be conducted for the design and development of suitable materials.

## 5 Concluding Remarks

To endow biomaterials with the capability to control cell behaviors in the context of biomimetics, we have developed simple and widely accessible methods to fabricate functionally graded surfaces. A variety of gradations, including physical components, mechanical modulus, and even surface structures have all been designed, fabricated, and applied to biological and biomedical applications, in particular, tissue repair or regeneration. Reinforced by surface gradations, scaffolds have been invented with enhanced bioactivity to yield promising outcomes in promoting neurite outgrowth, accelerating cell migration, mimicking native entheses, and related applications.

In addition to the methods discussed in this Account, there are also many other techniques for fabricating functionally graded surfaces and materials. Notable examples include the layered transition structures produced by liquid laminar flow in a microfluidic device,^51^ three-dimensional gradient scaffolding materials produced by layer-by-layer printing technology,^52^ and the Turing structures inspired by the “reaction-diffusion” mode in biological development.^53^ In particular, the Turing-patterned membranes, with tubular or vesicular nanostructures, increase water permeability by reducing reactant diffusion. These patterns, inspired by nature, can mimic the complexity of the extracellular matrix, promising potential use in the field of tissue regeneration.

Despite significant advancements in the development of innovative techniques for creating surface gradations, several challenges remain. These pertain to the diversity of the gradations and thus generality of the fabrication methods. Till now, the formation of gradients can be achieved through the utilization of a range of components, including inorganic and organic materials, and even cells. The significance of these components is contingent upon their intended application for a specific tissue or organ. The objective is to create a bionic structure that can closely resemble the autologous tissue, with the ideal outcome being a gradient that closely replicates the natural gradient observed *in vivo*. To this end, the methods involving one or two steps are advantageous in terms of reproducibility and processing efficiency.

The fabrication of multiple types of gradients on the same surface also presents a great challenge, as it necessitates the interaction between several distinct gradients. How to spatially combine the different types of gradations on the same surface has been the ultimate goal of essential research in the context of biomimetics. The current research priorities include the repair of tissue interfaces such as bone-cartilage and tendon-bone, as well as the development of multifunctional surfaces with osteogenic, angiogenic and neurogenic activities. For example, for the reconstruction of the tendon-to-bone enthesis, one should give considerable attention to the alignment changes of collagen fibers, the graded transition of mineral content, mechanical transfer between the soft and hard tissues, as well as the phenotypes of different cells. Furthermore, the advancement of wound repair materials has progressed from single-layered structures to multilayered ones. Nevertheless, the creation of bilayered or trilayered materials with epidermal to dermal gradients remains a significant challenge. Issues in multimaterial manufacture include resolution, interfacial bonding, and complex shape formation. Additionally, the long-term performance of gradient materials *in vivo*, especially under dynamic conditions, requires further investigation.

It is expected that the materials and methods discussed in this Account will inspire the readers to develop new strategies for fabricating functionally graded surfaces and even expand the scope of research to the three-dimensional system while pushing the use of graded surfaces toward clinical applications. To this end, exploring strategies capable of generating surface gradations with a wider variety of functional components is vital to the development of biological substrates and biomimetic scaffolds. It is particularly interesting to integrate topographical cues (e.g., aligned fibers, graded particles, secondary nanostructures) with mechanotaxis (e.g., gradation in stiffness or stress) presented by the supporting materials, in additional to the haptotaxis offered by surface-bound molecules (e.g., graded bioactive proteins) and the chemotactic cues enabled by the released payloads (e.g., biological effectors from core–shell nanofibers or microparticles).
